# Delivering social and public health programmes through community arms of professional football clubs

**DOI:** 10.1093/heapro/daaf106

**Published:** 2025-07-08

**Authors:** Jordan Maclean, Alice MacLean, Cindy M Gray, Stephanie Chambers, Craig Donnachie, Russell Jago, Kate Hunt

**Affiliations:** Institute for Social Marketing and Health, University of Stirling, Stirling FK9 4LA, UK; Institute for Social Marketing and Health, University of Stirling, Stirling FK9 4LA, UK; School of Social and Political Sciences, University of Glasgow, Glasgow G12 8RT, UK; School of Social and Political Sciences, University of Glasgow, Glasgow G12 8RT, UK; Department of Psychological Sciences and Health, University of Strathclyde, Glasgow G1 1QE, UK; Population Health Sciences, Bristol Medical School, University of Bristol, Bristol BS8 2PS, UK; Institute for Social Marketing and Health, University of Stirling, Stirling FK9 4LA, UK

**Keywords:** community arms, professional football clubs, social and public health programmes

## Abstract

Community arms of professional football clubs have become key third-sector players in mitigating health and social inequalities. This paper examines the factors affecting their capacity for delivering social and public health programmes in the community setting. Semi-structured interviews were conducted with 24 community staff members from the community arms of 22 professional football clubs and one non-professional club. This provided the basis for an interpretive thematic analysis which led to the development of three themes: ‘from football club community departments to charitable arms’, ‘the reach of community programmes’, and ‘challenges and opportunities of delivering social and public health programmes via community football club arms’. Charitable status has created more funding opportunities, enabling community arms to better prioritize community needs. The expanding reach of community programmes delivers social and health benefits to children, adults, and older adults (65 and over) from diverse cultural and socioeconomic backgrounds. However, numerous challenges and opportunities were reported as affecting community arms’ capacity to deliver these programmes. Safeguarding is a challenge, but partnerships offer an opportunity to address it. Co-dependency with the football club presents both challenges and opportunities for community arms. Staffing and facilities, funding applications and reporting on programmes, were all identified as challenges. Our findings highlight eight key recommendations specific to areas of oversight in the community arms of football clubs, including professional development, partnership working, board members, resourcing, funding, programme reporting, and conflicts of interest.

Contribution to Health Promotion

- Community arms of professional football clubs have increasingly established themselves as providers of social and public health programmes in the UK (and elsewhere) and are often able to engage populations that are otherwise considered ‘harder-to-reach’ in a range of health-promoting programmes.
- Charitable status has opened up some new avenues to accessing funding and can enable prioritization of needs in local communities.
- Community arms identified numerous challenges and opportunities in delivering programmes to improve health and social inequalities.
- We have developed a set of recommendations to help community arms overcome the challenges they face.

## INTRODUCTION

Professional football clubs have become key third-sector players in the UK and Scottish Government’s agendas for reducing health and social inequalities (Brown and Beacom 2023, Meir *et al.* 2024). The ‘hook’ of the professional football club has broad appeal for attracting and engaging men (Gray *et al.* 2011, Hunt *et al.* 2014), but it has also been shown to appeal to women (Bunn *et al.* 2018), particularly in terms of promoting positive health behaviour change and overall health and wellbeing (Hunt *et al.* 2013).

Numerous evidence-based social and public health programmes have been developed in the professional football club context. For example, weight management interventions have led to changes in a range of physical and psychosocial health outcomes (Gray *et al.* 2013, Wyke *et al.* 2019, Birch *et al.* 2022, George *et al.* 2022). Physical activity interventions have improved the quality of life of those living with cancer and in supporting desistance from crime of people serving community sentences (Rutherford *et al.* 2021, Newson *et al.* 2023). Football participation programmes support children and young people’s wellbeing and increase confidence of those with a disability or impairment (Robertson *et al.* 2018), as well as adults experiencing homelessness (Randers *et al.* 2019). Mental health programmes can increase social interaction, social capital, resilience, and confidence (Curran *et al.* 2016, Dixon *et al.* 2019, Wilcock *et al.* 2021, Llewellyn *et al.* 2022). Social inclusion programmes encourage belongingness amongst minoritized communities, including people of diverse religious and cultural groups, refugees, and asylum seekers (Maxwell *et al.* 2013, Spaaij 2015, Stone 2018, Taylor and Pringle 2022). Programmes also support difficult challenges in ageing societies, including living with dementia, being sedentary in later life, or living with an array of comorbidities (Carone *et al.* 2016, McEwan *et al.* 2019).

However, with the proliferation of programmes, it becomes even more important to understand the capacity of community arms in delivering social and public health programmes in the professional sports (football) club setting. Drawing on data from qualitative interviews conducted with community staff associated with professional football clubs across Scotland, this paper aims to examine the factors affecting community arms’ capacity to deliver social and public health programmes. This study extends previous work (e.g. Lozano-Sufrategui *et al.* 2020, Pringle *et al.* 2021) by including the perspectives of a more diverse group of community staff at varying levels of seniority, including chief executive officers, community managers, development officers, and trustees. Additionally, the Scottish context offers a unique contribution as it is home to the internationally recognized Football Fans in Training (FFIT) programme, which has been successfully scaled up and adapted in multiple countries (Hunt *et al*. 2020), making this a valuable case for international comparison and learning.

## METHODS

This study utilized in-depth qualitative interviews to explore the factors influencing community arms’ capacity to deliver social and public health programmes through professional football clubs. The project was approved by the University of Stirling’s General University Ethics Panel (16614).

Purposive sampling of clubs in all three divisions in the Scottish Professional Football League (SPFL) was facilitated by the SPFL Trust. The SPFL Trust is a third sector organization that supports community arms associated with SPFL clubs (*n* = 42) to reduce the impact of poverty through a number of strategic priority areas including health and wellbeing, and attainment (SPFL Trust 2022). To facilitate recruitment of club staff to the study, the SPFL Trust sent all community arms an invitational email which included the participant information sheet and an overview of the topic guide (see Supplementary File). Club staff interested in being interviewed were invited to contact J.M. by email. The SPFL sent one follow-up email to all clubs three months later. J.M. arranged interviews with all who responded. Fourteen clubs did not respond at all, four clubs showed initial interest but did not respond to follow-up emails, and one club declined to participate.

Twenty-four community staff (19 male/5 female) from 23 clubs completed an interview between May and August 2024. The interviewees held various positions within community arms, including five CEOs, nine community managers, seven development officers, and three trustees. They had varying levels of experience working at the clubs (mean number of years= 9; range 1–20 years). Community staff were chosen as they oversee the operations and delivery of programmes associated with the community arm. They represented community arms of clubs across all SPFL divisions: seven in the SPFL Premiership, five in the SPFL Championship, three in League One, seven in League Two, and one demoted to the Lowland League.

Semi-structured interviews lasted between 45 and 60 min. Interviews allowed community staff freedom to describe the opportunities and challenges they face within their own club context. All interviews were conducted online using Microsoft Teams, except one which was conducted in person. All but one participant had their cameras turned on whilst using Microsoft Teams. All interviews were audio recorded using a digital Dictaphone (Philips Voice Tracer DVT1160). The topic guide was developed based on the research aims and existing knowledge of the organizational structures of charitable arms. It was divided into four sections: (i) Charitable status, which covered the history of the charitable arm, staffing, and board of trustees; (ii) Current and past programmes delivered, which discussed the planning and organization of programmes delivered; (iii) Capacity, resources, and challenges community arms face in delivering programmes; and (iv) Sustainability and the future delivery of programmes.

The interviews were transcribed verbatim by a trusted transcription company. Selected transcripts were quality-assured by J.M., who listened to the audio recordings and compared them against the completed transcripts for accuracy. Our analysis was guided by an interpretive thematic approach, involving six stages that are grounded in identifying patterns across the interview transcripts (Braun and Clarke 2023). Once returned, stage one involved familiarization with the data. J.M. read through each transcript several times to check for inaccuracies and to familiarize himself with the data. Stage two involved generating initial codes. Members of the research team read through a selection of deidentified transcripts, and a coding framework based on short phrases, key ideas, and resonant concepts was developed by JM, discussed with the research team and then refined by JM. The coding framework consisted of both semantic codes—what interviewees said (e.g. where an interviewee talked about different types of community programmes, such as ‘education’, ‘health’, and ‘football’), and latent codes—hidden meaning or underlying assumptions (e.g. ‘co-dependency’ which is used to describe the dependencies between football clubs and community arms) (Byrne 2021). J.M. coded all of the transcripts using NVivo12. The resulting codes and sub-codes illustrate the nuance and depth across the interviewee responses prior to the development of themes (see coding framework in Supplementary material). Stage three involved searching for themes and sub-themes. Themes and sub-themes were generated deductively through an iterative process by grouping together overarching patterns in the codes. Stage four involved reviewing the themes in specific sections of the topic guide: the first section informed theme one; the second section informed theme two; and the third and fourth sections informed theme three. Stage five involved checking potential themes with the research team before they were finalized (Smith and McGannon 2018). Stage six involved identifying a selection of quotes to represent the themes which reflect variation in perspectives, including tensions or differing views between interviewees.

## RESULTS

This section presents three themes identified through the interpretive thematic analysis. The first theme ‘From football club community departments to charitable arms’ captures the reasons why football club community departments changed to become charitable arms of football clubs; the second theme ‘The reach of community programmes’ considers the reach of community programmes for meeting the needs of ‘hard-to-reach’ populations; and the third theme ‘Challenges and opportunities of delivering social and public health programmes’ identifies the challenges and opportunities of delivering social and public health programmes through community arms.

### From football club community departments to charitable arms

Before gaining charitable status, community departments were integrated within the organizational structure of the football club. However, there was a perception that the commercial side of the football club limited the community departments’ funding opportunities. For example, one interviewee said, ‘A lot of them [funders] were coming back and saying, you’re a limited company, this isn’t something we do, in order to become funded and for us to consider you, you need to be a charitable organisation’ (I5, Interviewee 5). There was also uncertainty over the funding that was raised by community departments, and whether it should ‘subsidise first team and youth team activities’ or go towards helping ‘people that needed that investment and support and impact’ (I4). Interviewees also felt clubs lacked knowledge of what the community arm was really doing: ‘they probably don’t fully know what we actually do in reality’ (I21). Another remarked on what they perceived as a lack of interest in wider activities: ‘I don’t expect ever to see a board of directors on the football club highly motivated about what’s happening within the charity’ (I6).

Most football clubs in Scotland now have a charitable arm [‘I think nearly every club, certainly senior club in Scottish football, […] nearly all have a charitable wing’ (I11)], but clubs varied in how long they had had charitable status, from 16 years to a couple of months. Several interviewees discussed their involvement in the process of gaining charitable status, which was described as ‘flip[ping] the football club model’ (I4) from community departments which were ‘originally … set up for football provision for kids’ (I9) to prioritizing the needs of local communities; as one said, ‘the need of the community is a lot greater than just a Saturday at three o’clock, or a young person playing football for the first time’ (I17). Interviewees described being proactive in identifying community needs [e.g. ‘I think you have got to look at what is the need, then go and try and meet those needs’ (I14)], which then underpinned the community arm’s strategic priorities and included broader aspirations such as alleviating poverty and improving health and wellbeing. One summarized the importance of health promotion within their community arm’s programmes, saying ‘ultimately, public health is what we are’ (I21).

The acquisition of charitable status has resulted in community arms becoming ‘a self-sustainable arm of the main club’ (I15) i.e. ‘structurally and constitutionally independent’ (I8). Consequently, community arms that are charitable organizations have their own board of trustees with a range of relevant expertise:

One whose background is in [profession], and he’s the Chairperson. He has been there since day one basically. We’ve got a finance [representative], for obvious reasons. We’ve got someone from a background in [Background Checking], the voluntary sector, Chief Exec[utive] of previous charities … Skills Development … mental health … sport and sports development … marketing and media … commercial support. (I21)

Interviewees generally viewed trustees as being ‘interested in the strategic side, like where are we going to be next year, where are we going to be in the next three years, is funding okay and stuff’ (I8). But the reported involvement of the board varied, from, ‘very passive’ (I13) to more active: ‘they’re [board] saying to me, that’s great, what about the other programmes? … how can we support you?’ (I14). Another suggested having a balance, ‘they could be more active, but actually if they are more active, that might be a hindrance’ (I18). Yet despite this apparently increasing separation and autonomy from the football club, several interviewees suggested that misconceptions about the function of community arms still prevailed. One said, e.g. ‘There’s a perception that [it’s] set up to feed the coffers of the football club when that’s not what it’s for’ (I1).

### The reach of community programmes

Clubs described a wide range of activities, mainly designed with the intention of supporting people who were perceived to need additional help because of their health, financial insecurity, or other vulnerabilities. At one club, community engagement involved working with minoritized ethnic groups from ‘a wide range of nationalities […] Afghanistan, the Sudanese, the Ethiopians, Ukrainians […] trying to help them get integrated within the [area] a little bit and explore, and it’s mostly targeting their mental health as well’ (I12).

Interviewees discussed the various educational programmes that they provided to support children’s attainment and expressed the belief that these ‘[take] so much pressure off teachers’ (I13) and allow parents ‘to work longer’ (I5). Children and young people had often been recruited via social media ‘because that’s where the parents will pick it up […] Facebook, it just works. You’re hitting parents’ (I6). Some also described reaching participants through ‘school apps and things, so parents can actually get just quick notifications about what’s available’ (I8). Interviewees described how they were able to reach out to support marginalized children and young people; one described supporting a significant number of secondary school pupils ‘that don’t interact, or find engaging with mainstream education hugely difficult’ (I4); others said their programmes were ‘dealing with kids that are disengaged at school, kids with all the kinds of backgrounds’ (I9) or helping to ‘support them to gain qualifications of some sort, where they might not get it in school’ (I16).

Food poverty programmes were also often talked about in the interviews, with one club saying they served ‘200 to 250 meals a week’ (I17). These focused on a range of populations: after school clubs were providing children with ‘a nutritious, healthy snack every week’ (I16) whilst another club provided meals for ‘the most isolated elderly citizens in our area’ (I3). Another had established a food hub service which aimed to minimize barriers for people needing support: ‘you get a bag with the [Football Club] badge on it, and you take the food, nothing asked, nobody knows where you are going’ (I5). One interviewee described how they tried to be sensitive to cultural differences: ‘Even in Match Day Experiences, we were taking folk over to the game, but it was during Ramadan […] I chose not to eat at the game, like I usually would, or drink, just to be a bit of an ally to the group that we were with’ (I16).

Several clubs ran attainment focused programmes which aimed to give ‘parents and guardians’ a chance to develop employability skills, such as ‘CV writing, job applying, and practising interview skills’ (I16), or help to support ‘young people aged 16 to 25 […] into a positive destination, into work, into a work placement, into even volunteering opportunities’ (I8). In a similar vein, an interviewee described one of their programmes as ‘a drop-in clinic with Citizen’s Advice’ (I3).

Another group which community arms aimed to support was people living with disability or neurodiversity. They described programmes which involved ‘kids aged ten to 16 who are autistic’ (I20) and ‘disability football for adults’ (I22). Due to increasing popularity, one charitable arm now provided programmes which ‘separate autism from para [disability sport]’ (I13); one said their football programme for people with autism reached ‘roughly 64 young people a week’ (I19), whereas another described the inclusion of disability sport as ‘the latest addition to our Foundation’ (I17).

The interviewees noted that many of their health and wellbeing programmes attracted men, including those addressing mental health issues; for instance, one said ‘on a Monday night, e.g. we open the stadium to men aged 18 plus to come together and talk and feel included, so… And that’s funded through a Health and Social Care Partnership under the suicide prevention department’ (I21). Other health-related programmes included supporting people’s mental health and wellbeing—sometimes geared specifically to particular age groups. One interviewee said, ‘We’re as well to go to some of the local groups working with older people, talk to them about what we’ve got and who do they have that might engage’ (I6). Programmes took various forms, such as ‘a walk and talk group and that is a mental health support group’ (I14) or ‘mental health football … in partnership with the NHS, and the Health and Social Care Partnership’ (I22).

Other programmes geared to other potentially vulnerable groups such as a ‘12-week course to support veterans that are struggling to adapt back into civilian life’ (I19). Some clubs had targeted particular groups of women, such as a programme for new mothers dealing with ‘postnatal issues, postnatal depression, just maybe anxiety, all these things that come along when you’re a new mum’ (I3), and a menopause group at another club, where they described holding sessions monthly:

Just once a month, and it is a two-hour session. The first hour … we go through a physical activity with the women, make sure they are still engaging in a physical activity, because we know that that can reduce symptoms of menopause. Then the second hour is just, like, a drop in café, so the women just get a little blether, a chit-chat, and then get, like, some refreshments as well. (I20)

Other programmes targeted men and women with specific health needs, such as a history of substance use. One interviewee mentioned a project which had reached ‘over 300 people […] over the course of a year. We’re comfortably getting 60 plus people engaging in it every week’ (I19). These programmes were seen as having extended a ‘lifeline’ to people who had attended:

I’ve had quite a few people come back to me and say, had they not got the help they got when they did, they’d be dead. And I’ve got, you know, people who are, have had addiction issues, alcohol. One chap who was an alcoholic, who is very open, and his life was just absolutely in tatters, you know. His family life was broken, and he was just, he was an absolute mess when he came to us, in all honesty. He has now been sober for two and a half years. He’s got his life back on track. (I3)

Some clubs reported people had continued to stay involved with community activities over several years after taking part in the specific programmes, such as the FFIT weight loss and healthy living programme (Gray *et al.* 2013, Hunt *et al.* 2013), originally developed for men, but now extended to women (Bunn *et al.* 2018): ‘We’ve had guys on the FFIT continuation that had been with us for the best part of eight years now’ (I19).

Social inclusion programmes were offered by several clubs as a way for ‘feeling part of something’ (I4). One had run ‘reminiscence’ programmes, which included a weekly football memories group for people ‘mostly with early onset dementia or Alzheimer’s, but some just living in loneliness and social isolation, they’ll come in and reminisce about football’ (I8). Another described a ‘Parkinson’s walking football’ (I14) group. Other programmes included a digital inclusion group which was ‘largely centred around social inclusion’ (I17), and social bonding was seen to be an important feature of other programmes:

[T]he walking football team spend longer in the café after [the game] having a tea and a coffee and talking and having that interaction with each other and that relationship that actually probably the walking football is now secondary to just coming out and having a chat with what they call friends. (I5)

Football participation programmes (i.e. where football is primary focus) were described as ‘historical’ (I22), as these programmes were predominantly delivered before gaining charitable status. Now that community arms are distinct entities more or less closely associated with their football clubs, one interviewee said they strategically separate football from their community programmes: ‘we are having one Community Club, which is the legal entity, which has two branches. One is the football, and one is the community foundation. That is only a branding exercise’ (I9). Nonetheless, football programmes are still popular for community arms and extended their reach to a large number of young people [e.g. ‘600 young people that come every week. From the age of 18 months to 20 [years]’ (I21)].

### Challenges and opportunities of delivering social and public health programmes via community football club arms

This theme includes four sub-themes that describe the challenges and opportunities involved in delivering social and public health programmes. ‘Safeguarding’ is a challenge, but partnerships with other organizations offer an opportunity for collaboration. ‘Co-dependency’ with the football club presents both challenges and opportunities to community arms. ‘Staffing and facilities, funding applications and reporting on programmes’ were all also identified as challenges.

### Safeguarding through partnerships

Coaches spoke of facing safeguarding issues when ‘dealing with some really challenging people or people that have real challenges in their lives’ (I19). Some interviewees believed that coaches ‘are lacking in a lot of necessary […] people skills’ (I11). They suggested that there was a requirement for additional workplace training so that those working in the community arms of clubs would be better equipped to pick up on the needs of some of their clientele, so they could better read people’s behaviours and ‘look for signs’ (I3). They gave examples of how this training could be put in place, including through partnerships with other organizations which provided coach training in specialist areas, such as disability coaching:

[A national disability charity] will come out and give training to the coaches who are going to be doing delivery of it [programme] … it means that we’re doing it right and we’re understanding the needs of the people that are actually on there. (I1)

Interviewees showed awareness of their responsibilities and expressed an expectation of the need to safeguard participants. As one said, ‘So, anybody that comes through our door, we want to ensure that they go out a better version of themselves or an improved version of whatever aspect they want to get out of it’ (I5). Interviewees were also conscious of the need for safeguarding for staff, both for their own benefit and for those taking part in their programmes. One said, ‘I just did everything, and you burn out very, very quickly […] if I am not looked after I am not going to be able to be appropriately looking after those that I am supporting’ (I14).

Partnership working was described as essential by some interviewees ‘Everything we do, we do in partnership with other organisations’ (I3), but others had no current partnerships in place. Some interviewees felt there was the need for more collaboration between community arms. One said, ‘I think a lot of foundations and trusts need to be working together a lot more because, you know, there are projects that we run that maybe other trusts [community arms] don't run, and vice versa’ (I20). However, another stressed the importance of finding the ‘right’ partners, ‘I think you need to pick the right partners and ensure we’re protected in Community Football Club and the programmes are protected’ (I21).

### Co-dependency with the football club

There was a sense from the interviews that the football clubs and their community arms are co-dependent, as conveyed here: ‘So, if the football club is not here, the community doesn’t exist and if the community doesn’t exist, then the football club doesn’t have as many supporters’ (I5). There was also a sense that the relationships between the two entities were somewhat fluid and sometimes needed renegotiating. One interviewee described how they present their position with the club, explaining that they were ‘a separate legal entity when it suits us, and we are not a separate legal entity when it does not’ (I8). Several said that having a trustee from the football club involved in the community arm helped to maintain good partnership working between the club and its charitable arm, which was described as ‘vital to the success of the charity’ (I8), and helpful in other ways such as providing match-day tickets, use of their stadium and ‘get[ting] access to players now and again’ (I19). However, one community arm no longer had a trustee from the football club on their board because the discussion primarily focused on the performance of the football club, ‘If you asked me at that time [when they did have a board member from the football club] that question, I would say, pointless, [I]t’s a waste of time. We go to board meetings. We speak about Football Club’s results’ (I21).

Many were aware of the power of the football club brand ‘the club badge really captures people’ (I19) and that their reach would not be as great without it. One said: ‘I think we would find it hard to have the same influence and footprint’ (I14). However, they also recognized a risk that the branding of the club could overshadow the work of the community arms, or that they would be perceived as one and the same: as one explained, ‘once you are within the Club branding, although you’re a separate entity, and it makes you want to tap into that, you’re at risk of being, you are [Football Club]’ (I8). Some had created a separate logo to differentiate themselves from the football club. One said, ‘It is just that we want to split that football side of things up … It is just a new name on it, but some way of going, ‘this is separate, this is new’’ (I9).

Interviewees were conscious that the fortunes (and reputation) of the club could fluctuate, particularly in response to their team’s standing. Interviewees noted that when teams were performing less well, the club could draw on the success of community work:

[T]he press and the attention we get from the Football Club is an interesting one around our projects. So, it could be the instance where your Football Club has won six games in a row, [the] chances of us getting any shining light on our work, probably very slim. [If] the Club has lost six games in a row, needs a good news story. Very much so. Let’s get the feelgood back. Let’s get the fans back on side. Let’s make the Club, you know, position in a way that there is a positive feel to it. (I17)

The changing fortunes of the clubs could have particularly notable effects if they were relegated from or promoted to the SPFL. One interviewee noted a club’s relegation from the SPFL would have ‘a bounce effect for the community trust’ (I11), as it would exclude them from SPFL Trust activities and support. Another noted the benefits of a club’s promotion:

[I]n the last, you know, year since they’ve been promoted […] has allowed the foundation to really, kind of, use that as a higher level than what it was when they were playing the lower league, because it has a greater status. (I4)

### Staffing and facilities

There were differences between the staffing of community arms in smaller and larger clubs. Community arms of larger clubs tended to have more full-time members of staff (sometimes more than 20), whereas smaller clubs were reliant on one or two staff members, which limited what they felt able to take on: ‘I’m, kind of running and driving all these programmes. So, I have to keep things to a minimum’ (I3). Furthermore, one interviewee said that the COVID-19 pandemic had made it more difficult to recruit volunteers to support their activities, ‘We really struggle now to get volunteers. I think COVID killed, COVID killed the concept of having people volunteering in communities’ (I3), whilst another felt uncomfortable about relying on volunteers: ‘I don’t also like using volunteers because right now it’s a cost-of-living crisis […] who wants to do stuff for nothing in this day and age? Do you know? It’s so unfair’ (I10).

In addition to some precarity with staffing, the physical space available within the stadia could present problems, including inadequate office space for community arm staff. Few community arms had their own facilities, and when they did, they were sometimes able to use this as another revenue stream (e.g. by charging others to use their facility) that they were then able ‘to reinvest back into our social impact programmes’ (I4).

Community arms who shared facilities with their football club had to work with their club as the gatekeeper to facilities, fitting around football fixtures and other club events. Some described needing to pay for facilities, whereas others had access to facilities for free, and some had to rely on local authority facilities.

### Funding applications and reporting on programmes

The funding landscape was described as ‘an ever-competitive landscape that is probably getting more and more difficult to navigate’ (I17). Short-term funding was most prevalent: ‘Nothing is long-term […] But this, kind of, constant piecemeal short-term approach’ (I6), and some clubs were limited in what funding they could apply for, either because of the size of their club or because of the (lack of) experience and expertise of their staff. Many interviewees described feeling ill-prepared to write funding applications ‘I’m not an expert in writing funding bids […] But there’s not anybody else, so I do it’ (I3) and felt better able to approach funders in person ‘If I could go face-to-face with somebody and tell them why this programme needs to run, I would storm it’ (I10). A few said they did have the right expertise for writing funding applications: e.g. one said, ‘my career in [my previous job], working with people, relating to people, being organized, being able to write policy statements, write bids for funding and so on, so it has been very, very useful’ (I11). Others in larger clubs relied on business managers or funding agents.

One interviewee was strategic in using the name of the football club to attract funding:

I would say, certainly, above 50 per cent, but maybe about two-thirds of our applications, we will apply with the term, [Charity name]. And we won't even use the term, [Football Club], as a primary aspect […] But if we’re doing, you know, an anti-poverty programme, and there's even some aspect of a funder’s unease, or a community unease, we are not so precious about needing credit or needing to take claim of that. (I16)

However, there was a sense that community arms are applying for a finite number of funding calls, ‘Everybody’s got the same directions. Everybody’s got the good stories to tell. But there’s only so many people that you can get the funding from’ (I18). Some interviewees mentioned how this might lead to diversifying their funding portfolio in order to ensure the sustainability of their programmes ‘You don’t want price to be a barrier [to participation]. So yes, I guess, there is going to be a gap there and that gap has to be filled with funding, fundraising, or corporate sponsorship’ (I22). Another said, ‘we’ve got to, kind of, fight tooth and nail to get, you know, pockets of money in the door. That’s probably why we’ve broke into the corporate route more’ (I17).

The need to report back on programmes was described as ‘an ongoing process’ (I17), including to the board of trustees and funders. It varied from ‘very light touch […] [to] very, very detailed reporting’ (I11): ‘so you have to do X, Y and Z’ (I9); ‘[some funders] need all the numbers, they need case studies, they need everything to justify the spend […] [whereas others] have a phone call once a month’ (I5). Nevertheless, interviewees needed to provide relatable feedback on their programmes, ‘Numbers aren’t enough anymore […] but see the case study of little Johnny who is now confident enough to go and do X, Y and Z, […] that lights a fire a lot more’ (I9). However, one interviewee also highlighted the need for greater surveillance to ensure improved accountability, ‘I think we should be critical of ourselves, even though no one likes to be inspected, or audited […] [if] […] we are doing it poorly, we deserve to be told that’ (I16).

## DISCUSSION

Our findings show the wide range of potentially health promoting initiatives undertaken by the community arms of professional football cubs in Scotland, despite increasing social challenges precipitated by the Covid-19 pandemic and a cost-of-living crisis. Gaining charitable status has opened up more funding opportunities to prioritize these community needs. The expanding reach of community programmes brings social and health benefits to hard-to-reach populations. However, numerous challenges and opportunities of delivering social and public health programmes were reported by community arms.

In Scotland and elsewhere, gaining charitable status has created legal separation between football clubs and their associated community arms (Anagnostopoulos *et al.* 2022). Our interviewees described charitable status as ‘flipping’ from football-focused community departments to charitable organizations to support a broader health and wellbeing focus. A key reason for doing so was because football clubs are commercially focused, while community arms are community focused. In other words, the ‘profit maximization’ of football clubs can be perceived to be at odds with the altruism of community arms (Anagnostopoulos *et al.* 2017). According to Walters and Chadwick’s (2009) analysis of the governance structures of two English football clubs’ charitable arms, ‘any funding received [e.g. government or commercial] has to be spent on developing community activities and cannot be used by the football club’ (p. 60). Our interviewees also said they now have greater access to public funding for social and public health programmes. This is consistent with Hamil and Morrow’s (2011) findings, where interviews with football club officials revealed that separate governance structures make it easier for charitable arms to access public funds. Nevertheless, while their strategic priorities address community needs, a misconception persists regarding the financial security of community arms due to their association with football clubs. However, this is not the case; indeed, Bingham and Walters (2012) have argued that many clubs provide little financial support, if any, to community arms.

The expanding reach of community programmes brings social and health benefits to hard-to-reach groups including underrepresented populations. Rowe *et al.* (2018) found that community programmes of professional sport teams are strongly aligned with government policy areas (e.g. health, education). However, according to Sanders *et al.* (2020) diversification of programmes has resulted in the centrality of football activities decreasing in importance. Programmes discussed in this study target both men and women, and minoritized or marginalized groups such as refugees, older people, people with disabilities or specific health conditions, and those with a history of substance use. Yet, it is important to note such diversification of target groups is not typical of football clubs in Europe which tend to prioritize children and youth, rather than adult, populations (Lozano-Sufrategui *et al.* 2020, Røynesdal *et al.* 2021).

Our study highlighted numerous challenges and opportunities for delivering social and public health programmes through the community arms of professional football clubs. While partnership working has already been identified as crucial to community arms (Misener and Doherty 2013, Pringle *et al.* 2021), some interviewees found it difficult to establish partnerships. Our interviewees also recognized the importance of safeguarding for their staff and volunteers. Cronin and Lowes (2019) noted that whilst safeguarding is covered in coach education programmes, its focus is often limited. With increasing social responsibilities (Van Der Verken *et al.* 2019, Smith *et al.* 2021), it is even more important that coaches, who are often volunteers or paid staff in precarious work (Ives *et al.* 2019), receive safeguarding training that is appropriate for those participating in their programmes (Partington 2014, Gurgis *et al.* 2022). Interviewees also mentioned the difficulty of recruiting volunteers in post-pandemic times. This is despite evidence which suggests that some community arms in Scotland expanded their staffing during the pandemic (Oeckl and Morrow 2022).

The ‘co-existence and interaction’ (Hyndman and Liguori 2023) between the charitable arm and the football club means that their organizational and activity boundaries are frequently blurred (Anagnostopoulos *et al.* 2022). Interviewees highlighted the potential for blurring of organizational boundaries when having a trustee from the football club on their board. Others noted the blurring of activity boundaries when clubs provided community arms with players, match-day tickets and use of their stadium.

Our study also demonstrated that football clubs can draw on the success of the work of charitable arms. While doing so can boost the clubs’ profile and reputation (Walters 2009), ‘turbulent’ periods for the first team can have negative implications for community arms (Adams *et al.* 2017). Relegation might mean that ‘institutionalized’ (Anagnostopoulos *et al.* 2017) funding and support (e.g. through the SPFL Trust) is no longer available to community arms of clubs that fall outside of the main leagues. However, some interviewees mentioned the community arm and the football club can get a publicity boost when the club team is not playing well.

There is increasing accountability imposed on community arms to report on the social and health impact of their programmes (Harris and Adams 2016, Davies *et al.* 2020). While our interviewees discussed wide variation in funders’ reporting structures, individual stories (case studies) were identified as a potentially powerful way for evidencing impact. However, Brazier *et al.* (2023) found that few professional sports clubs in England report on the impact of their activities on participants’ health and wellbeing, and those that do often relied on evaluations that were anecdotal and used self-reported data.

Some interviewees mentioned that they lacked the expertise needed for writing funding applications, highlighting the need for further training and guidance. Anagnostopoulos *et al.* (2014) recommend including external board members with strong local standing and/or relevant expertise. These individuals can be a valuable resource for community arms, especially in tailoring funding applications to address social and public health issues that local authorities and governmental agencies aim to tackle.

As public funding is constrained, some interviewees mentioned they are diversifying their funding portfolio by focusing on corporate sponsors to ensure the future sustainability of their programmes. Previous research has identified corporate social responsibility as an area of growing interest in community arms of professional football clubs (Bingham and Walters 2012, Bostock *et al.* 2021). However, the ‘community value’ (Trendafiova *et al.* 2016) of corporate citizenship must be weighed carefully, because there is the potential for major conflicts of interest between health promotion and unhealthy commercial sponsorships (Ireland *et al.* 2024).

Our findings highlight eight key recommendations for oversight bodies (such as the SPFL Trust) in supporting community arms of professional football clubs (see Table 1). Areas for oversight include professional development, partnership working, board members, resourcing, funding, reporting on programmes, and conflicts of interest.

**Table 1.** Key recommendations for oversight bodies supporting the community arms of professional football clubs.

Areas of oversight	Recommendations
Professional development	1. ‘Providing programme delivery and safeguarding training or signposting staff to relevant resources, ensuring all those working in community arms are better equipped to support themselves and meet the needs of participants’.
Partnership working	2. ‘Establishing and building partnerships with organizations that can provide training for coaches in specialized areas, such as disability coaching’.
	3. ‘Fostering collaboration between football club community arms to share insights into community programmes’.
Board members	4. ‘Appointing board members with diverse expertise that aligns with community arms’ social and public health programmes to ensure a well-rounded and effective governance structure’.
Resourcing	5. ‘Proactively assisting smaller community arms with limited staffing or facilities by prioritizing the recruitment of volunteers and providing logistical support to strengthen their operations and ensure long-term sustainability of programmes’.
Funding	6. ‘Signposting community arms to funding opportunities and providing support with writing funding applications, especially for smaller clubs’.
Reporting on programmes	7. ‘Providing support and training for reporting to enhance accountability to the board of trustees and funders’.
Conflicts of interest	8. ‘Informing and advising community arms about potential conflicts of interest when diversifying funding to include corporate sponsorship’.

Our study has strengths and limitations. A key strength is interviewing 22 professional football clubs, representing over half of all SPFL clubs in Scotland. Participants also reflected a range of roles and levels of seniority within the community arms of sports organizations, including CEOs, community managers, development officers, and trustees. In terms of limitations, recruitment posed a challenge. Fourteen clubs did not respond to the study invitation, and it is unclear whether they did not receive the invitation or actively chose not to participate. Additionally, while the study focuses on professional football clubs in Scotland, the findings and recommendations have relevance for sporting organizations in other countries and other sports (see e.g. Hunt *et al*. 2020) looking to gain charitable status in order to deliver social and public health programmes. However, the transferability of findings to other sports or countries may be limited by whether or not clubs have charitable status.

## CONCLUSION

This study examined the factors affecting the capacity of community arms of professional football clubs to deliver social and public health programmes in Scotland. The findings highlight several challenges and opportunities in delivering these programmes within the community setting. We identified eight key recommendations for oversight bodies supporting community arms of professional football clubs, which are also relevant for other professional sporting organizations delivering social and public health programmes.

## Supplementary Material

daaf106_Supplementary_Data

## Data Availability

The data underlying this article will be shared on reasonable request to the corresponding author and the study principle investigator.
