# Supplementary material for: Delivering social and public health programmes through community arms of professional football clubs
Source: Health Promot Int. 2025 Jul 8;40(4):daaf106. doi: 10.1093/heapro/daaf106 (PMC12235519; doi:10.1093/heapro/daaf106)
Supplement: daaf106_Supplementary_Data [file daaf106_supplementary_data.zip › HPI Coding Framework.docx]

**Fitter Families**

Codes and subcodes

| Name | Description |
| --- | --- |
| Charitable status |  |
| Community focus | Responsibility to support local community ‘needs’. Strategic priorities (e.g., alleviating poverty, improving health, inclusion) are different to commercial focus of the football club. Still remains a misconception (and stigma) of being football focused in name and branding. |
| History | Temporality of the transition from community department to charitable arm of club (including changes over time and shift in focus, ethos) and shift from football/youth and education to wider initiatives. |
| Reliance/Independence from club | There was a reliance on clubs in terms of broader engagement, access to players, tickets. Charitable status meant they were ‘structurally and constitutionally independent’ from the football club. Knock-on effect if smaller clubs are relegated to lower leagues that fall outside of SPFL. Poorly performing clubs can draw on success of community work. |
| Community programmes | Community engagement, disability, education, employability, food poverty, football, health and family support |
| Facilities | If shared facilities with club, access is dependent on first teams’ schedule. Small number have their own facilities. Others are ‘nomadic’ in the sense that they use local authority facilities. |
| Recruitment and marketing | Branding of club is powerful. Different methods (e.g., social media, apps, word of mouth) for targeting certain population groups (e.g., children, parents, older people, receiving referrals from partners). |
| Funding |  |
| Applications | Many feel ill-prepared to write funding applications. Learning which pots of money to go for, how to write a good application = more success. Having expertise through staffing/trustees to be successful. |
| Cost to run v. sustainability | Charging may still be required (despite being non-profit), but it is then reinvested back into programmes. Longer funding periods would be preferred. Constant need to look for funding, bringing in staff on zero hours contracts has impact on staff retention and having really well-trained staff. |
| Partnerships | Partnership working is crucial to deliver on outcomes that have impact. Finding the ‘right’ partners who share the same values is important. |
| Governance |  |
| Board of trustees | Contribution of trustees varied. Specialised knowledge of trustees was strategically important to influence the priorities of the charitable arm. |
| Reporting | Reporting is ‘ongoing’ with funders, board of trustees and Scottish Charity Regulator, OSCR. Reporting criteria varied from ‘light touch’ to ‘very detailed’. Evaluation was built-in to programmes, but it needs to be approached in different ways for different funders (e.g., the power of case studies vs quantitative evaluations). |
| Staffing | Concerns raised about retaining paid staff and attracting new volunteers. Smaller clubs struggled with recruiting suitable paid staff, while larger clubs lose younger staff to organisations that can offer full-time roles. Most rely on volunteers but have noted difficulties in recruitment since the pandemic. |
