# Supplementary material for: Delivering social and public health programmes through community arms of professional football clubs
Source: Health Promot Int. 2025 Jul 8;40(4):daaf106. doi: 10.1093/heapro/daaf106 (PMC12235519; doi:10.1093/heapro/daaf106)
Supplement: daaf106_Supplementary_Data [file daaf106_supplementary_data.zip › Supplementary file.docx]

**Challenges and opportunities for delivery of public health interventions in professional football clubs**

Topic Guide for community coaches/managers

**Questions in this topic guide are to be used as a guide and not a script. Questions can be adapted by interviewer according to the flow of the conversation and the topics raised by the participant.**

Thanks for agreeing to take part in this interview to help us find out about the challenges and opportunities you come across in delivering public health interventions at [Name of Football Club/Community Trust]. The research is funded by the Chief Scientist Office and we are working in collaboration with Universities of Glasgow, Strathclyde, and Bristol and in partnership with the SPFL-T.

We expect the interview to last about 45 minutes (depending on what you have to tell us). We will discuss your own experiences of delivering public health interventions at [Name of Football Club/Community Trust] or any other football club; what helps you deliver them; what makes it more difficult; how things have changed over time (and why); and how you see the future for the delivery of these programmes at your club/foundation.

In advance:

- Check consent form completed.
- Explain that we are interested in personal views – not asking you to speak on behalf of your organisation – and that your name and that of your organisation will be de-identified in any reporting e.g. as ‘community coach’ or ‘community manager’. It is important you feel comfortable speaking openly and freely so that we get the best possible understanding of your views.
- Ask if any questions.
- Check if okay to record, then TURN ON RECORDER before starting.

1. Introduction
2. Our role in conducting the interview.
3. Please could you introduce yourself and your role at [Name of Football Club/Community Trust].
4. How long you have worked as a manager/coach at [Name of Football Club/Community Trust], and any similar roles you have had at other clubs.
5. Can you tell us about [Name of Football Club/Community Trust]? Prompt: size, staff, how long it has been established; does it have charitable status?
6. What facilities do you use? Do you use spaces inside the club or elsewhere?
7. Can you talk me through your weekly programmes (ie, Monday to Sunday) that are delivered in the club/trust?

How do these get up and running (i.e. funding, collaborations) and what is your role in that process?

Prompts: target population; target problem; partner organisation (apart from SPFLT)

Any they have run in past that they had to stop at this funding stage – reasons for not continuing?

1. How do you recruit participants for your public health interventions?

What strategies do you tend to use? (prompt if not forthcoming: social media; word of mouth; marketing via the club)

What strategies work well/less well?

How do you apply them for different programmes?

What has uptake been like on these programmes?

Any they have run in past that they had to stop at the recruitment stage – reasons for not continuing?

1. Thinking of the delivery stage - What have you found helpful in terms of support needed to deliver them? (ask about recently and previously)

Prompt: Support from SPFLT, support from club, support from other people/organisations, training, use of facilities, FFIT reporting portal – probe each of these if mentioned

How have these changed over the last 10-15 years? Why?

1. Thinking of delivery - What challenges have you experienced in delivering them? (ask about recently and previously)

Prompt: Support from SPFLT, support from club, support from other people/organisations, use of facilities, FFIT reporting portal, recruitment, maintaining participant engagement – for each challenge ask how they deal/have dealt with them

How have these changed? Why?

1. Thinking of the stage after programmes have finished – What happens? Who do you report to? And how well is this managed?
2. Thinking about the future now – what do you think are the most important resources, support and collaborations you need to sustain successful deliveries of public health interventions going forward?

How confident are you that you will get these resources, support and collaborations? Why do you say that?

1. Still thinking about the future – what concerns do you have about being able to sustain successful deliveries of public health interventions going forward?

What would help to address these concerns?

1. We are currently piloting a Dads and Kids healthy lifestyle and relationship building programme at two SPFL clubs, with the aim of rolling it out to other clubs if it is successful.

The programme was developed by Prof Phil Morgan at the University of Newcastle, Australia. HDHK is a 9-week programme that consists of one Dads only information session, followed by eight D&K sessions. Each week consists of a 30-minute classroom session on topics such as screen time and healthy eating, followed by 60-minutes of activity including rough and tumble play, sport skills, and fitness.

Is this something you would be interested in delivering? Why do you say that?

Prompt – opportunities/challenges of delivering HDHK

1. Is there anything else you’d like to add, or anything you’d like to bring to our attention that we might not have asked about?
